# Effect of snail mucus on angiogenesis during wound healing

**DOI:** 10.12688/f1000research.51297.1

**Published:** 2021-03-05

**Authors:** Yosaphat Bayu Rosanto, Cahya Yustisia Hasan, Rahardjo Rahardjo, Tri Wahyu Pangestiningsih

**Affiliations:** 1 Oral and Maxillofacial Surgery, Faculty of Dentistry, Universitas Gadjah Mada, Yogyakarta, Indonesia, 55281, Indonesia; 2 Anatomy, Faculty of Veterinary Medicine, Universitas Gadjah Mada, Yogyakarta, Indonesia, 55281, Indonesia

**Keywords:** new vessels, hematoxylin eosin, CMC-Na, glycosaminoglycans, heparan sulfate

## Abstract

**Background:** Angiogenesis is the process through which new blood vessels are formed from existing ones. This process plays an important role in supplying the oxygen and nutrients needed for cellular metabolism and eliminating cell debris during wound healing. Snail mucus can bind to several factors that stimulate angiogenesis, including vascular endothelial growth factor, platelet-derived growth factor, and fibroblast growth factor. The aim of this study is to observe changes in angiogenesis during the healing of wounds topically applied with snail mucus.

**Methods:** Punch biopsy was performed on the back of male Wistar rats to obtain four wounds, and different concentrations of snail mucus were applied to each of these wounds. The animals were sacrificed on days two, four, and seven to observe the extent of angiogenesis during wound healing by microscopy.

**Results:** Two-way ANOVA showed differences in number of blood vessels formed (p = 0.00) and day of observation (p = 0.00) between groups. Post hoc Tukey’s honestly significant difference test showed that 24% snail mucus treatment does not significantly affect wound healing (p = 0.488); by contrast, treatment with 48% and 96% snail mucus demonstrated significant effects on angiogenesis (p = 0.01). Spearman’s test showed interactive effects between snail mucus concentration and day of observation on the extent of angiogenesis (p = 0.001, R = 0.946).

**Conclusion:** Topical application of snail mucus gel can increase angiogenesis during wound healing in Wistar rat skin.

## Introduction

Oral surgery is an aspect of dentistry that is often associated with skin lesions ^1, 2^. Cuts to the skin during oral and maxillofacial surgery may be achieved through incision or excision. Incision wounds are usually established during surgery, while excision wounds occur in trauma cases ^3– 6^. Excision wound models generally have a diameter of 2–20 mm. Excision wounds provide complex and detailed views of the wound healing process and allow the examination of various wound healing parameters ^7^. This fact underlies the selection of excision wound in the present study.

When a wound occurs in the body, physiological healing is performed by multiple biocellular and biochemical processes ^8^. Wound healing refers to the process through which normal tissue is regenerated from damaged tissue ^9^; it involves cells, the extracellular matrix, and a number of mediators, such as growth factors and cytokines ^10^. The wound healing process also involves hemostasis, regeneration of peripheral cells, and restoration of muscle tissue by collagen fibers ^11^. Wound healing is a dynamic and complex process that involves multiple phases with overlaps from one phase to another ^12^.

The wound healing process can be divided into three phases, namely, inflammation, proliferation, and tissue remodeling. This process can be observed using several parameters, such as re-epithelialization, number of polymorphonuclear leukocytes, number of fibroblasts, density of collagen fibers, and angiogenesis ^13^. Angiogenesis is important in healing and refers to the process through which pre-existing blood vessels generate capillary buds to produce new blood vesselss ^14^. Angiogenesis is triggered by tissue damage, which causes local hypoxia ^15^. When local hypoxia occurs, cells respond by increasing their production of vascular endothelial growth factor (VEGF), one of the most important mediators for wound healing and a stimulant of capillary growth. Angiogenesis is then induced to fulfil requirements for nutrients, oxygen, and inflammatory cells ^16^.

Wound healing can be enhanced by chemical and natural treatments ^17^. Traditional medicines with natural health benefits and limited side effects have been developed by many researchers ^18^. Snail mucus is widely used by cosmetics manufacturers as a skin care material ^19^. The resulting products usually feature high contents of hyaluronic acid, proteoglycans, glycoprotein enzymes, and antibacterial peptides to protect the skin from damage ^20^.

The African giant snail ( *Achatina fulica*) contains glycosaminoglycans ^21^. Indeed, approximately 3%–5% of the dry weight of the snail is composed of glycosaminoglycans ^22^. Glycosaminoglycans are a group of anionic polysaccharides that are typically isolated as proteoglycans connected to protein nuclei ^23^. The biological activation of glycosaminoglycans stimulates the regulation of cell growth via the interaction of the glycosaminoglycan chains with growth factor proteins and their receptors ^24^. The snail also contains acharan sulfate, which is stored as granules in the snail body and secreted by the animal under certain stimuli ^25^.

## Methods

### Snail mucus gel preparation

The rat cages measured at least 40 cm long, 15 cm wide, and 10 cm high, and one cage housed one rat. The cages were covered with rice husks to achieve a stress-free environment for the rats, and the animals were provided food and water ad libitum. Snails are obtained from farms in Central Java. Identification and determination of Achatina fulica species were carried out in laboratory of animal biology. The snails were adapted to a cage with moist soil and banana leaves as food for three days. Then, the snails were fasted three days before the mucus extraction. Snail mucus was extracted by stimulating the surface of the snail body with an electric shock of 6 V for 60 s, one touches namely from repetition 2 ~ 4 times. This method does not cause pain or stress on the snail. A detailed description of all protocols can be found in the google patent numbered CN102846519B ( https://patents.google.com/patent/CN102846519B/en). The collected snail mucus was passed through batiste cloth to remove impurities, collected in a glass beaker, and homogenized. The snail mucus was sterilized by filtration through Whatman No. 4 filter paper. Finally, the mucus was diluted with CMC-Na to obtain gels with mucus concentrations of 24%, 48%, and 96% (w/w).

### Animals and group preparation

This research was approved by the Health and Medical Research Ethics Committee of the Faculty of Dentistry, Universitas Gadjah Mada (Approval No. 00272/KKEP/FKG-UGM/EC/2019). This study used nine rats based on calculations using the resource of equation method with the minimum sample calculation formula in research with ANOVA design. Sample calculation: Minimum n= 10/kr +1 =10/(4x3) +1 = 1.83. Maximum n= 20/kr +1 = 20/(4x3) +1 = 2.66 (k = number of treatments, r = number of repeated measurements). The conclusion is the use of 3 rats meets the sample size requirements.

The rats were obtained from breeding by laboratory of pharmaceutical. Healthy male Wistar rats were adapted to cages for 3 days. The rat cages measured at least 40 cm long, 15 cm wide, and 10 cm high, and one cage housed one rat. The cages were covered with rice husks to achieve a stress-free environment for the rats, and the animals were provided food and water ad libitum.

### Animal treatment

**Figure 1.**
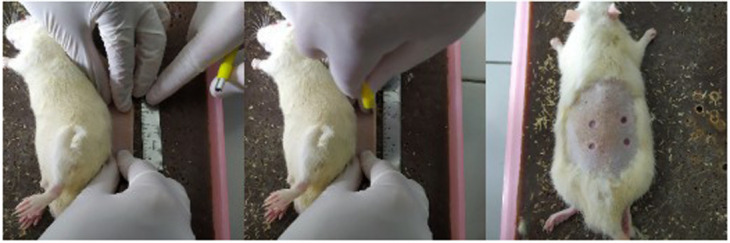
Installation of excision wounds.

**Figure 2.**
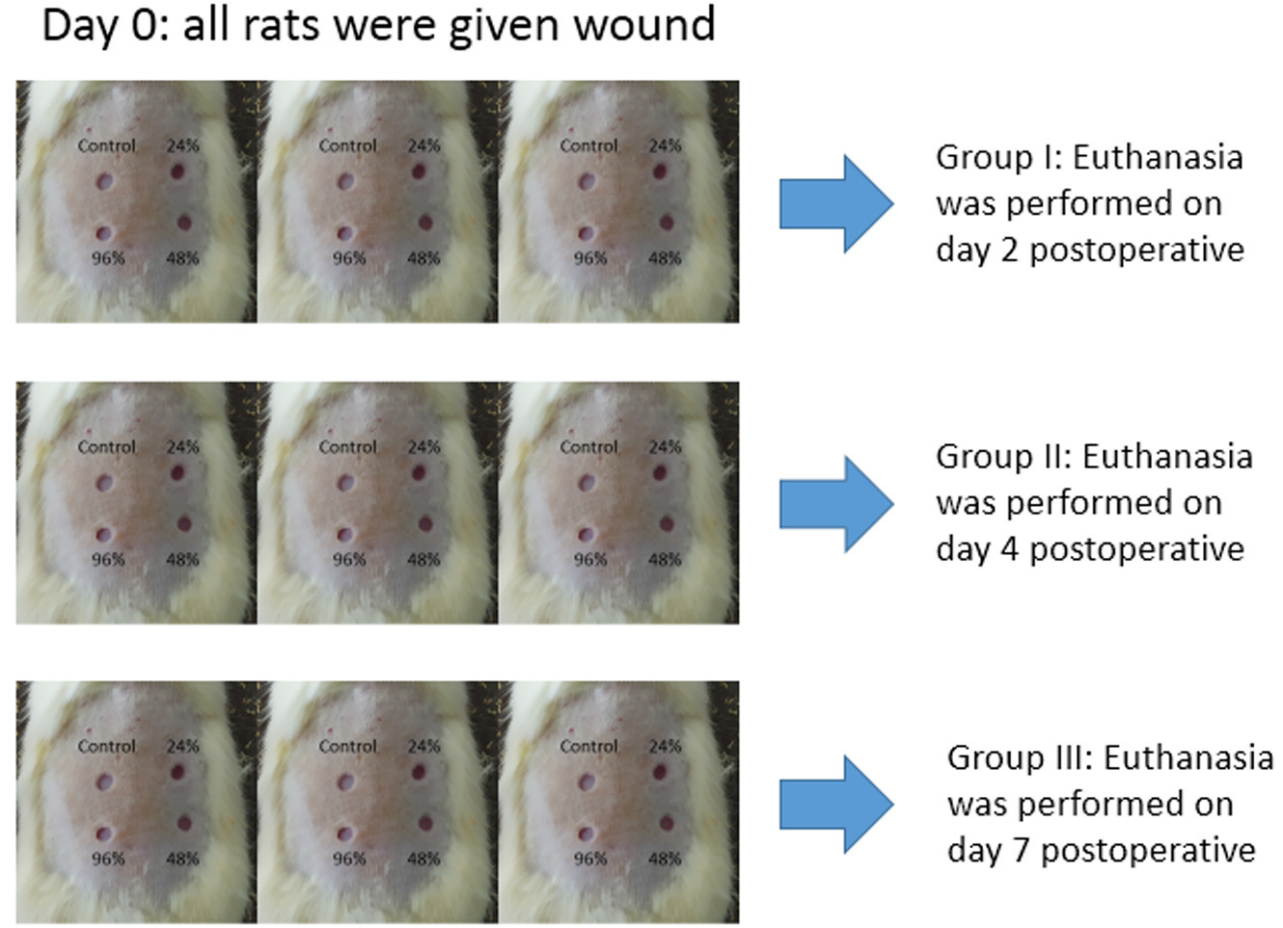
Experimental groups.

The rats were included in the study if they were 3–4 months old, weighed 250–300 mg, appeared healthy and physically active, and there were no visible anatomical defects. The rats were excluded in the study if they had postoperative infections or died before the euthanasia process. They were randomly divided into three groups of euthanasia day (three rats/group). Random numbers were generated using the standard = RAND() function in Microsoft Excel. The rats were anesthetized with 100 mg/kg BW ketamine and 4 mg/kg xylazine intramuscularly. The back of each rat was shaved, marked, and disinfected with 70% alcohol. A circular subcutaneous excision wound was made by punch biopsy of 5 mm. The skin on the back of a rat was folded and lifted by pinching the cranial and caudal skin between the thumb and forefinger. The rat was placed in the lateral position, and a biopsy punch was made through the folded skin (middle). Appearance of the resulting symmetrical and full-thickness wounds is shown in Figure 1. The minimum distance between wounds was 5 mm. Each rat was given 4 wounds which were given different experiments (24%, 48%, 96% and control gels), so the total was nine rats with 36 wounds (each experiments were nine wounds which were observed on day two, four, and seven). The total rats in each groups are described in Figure 2. The calculation of the number of samples and four wounds on the back of the rats and this fulfills the requirements of reduce, reuse, and recycle.

The rats were transferred to a warm cage until they regained complete consciousness and then returned to their original cages. The general condition and weight of the rats were recorded daily. The snail mucus (24%, 48%, 96%) and control gels (CMC-Na) were applied 1 ml once a day (in the morning) on each wound. Euthanasia was performed on postoperative days 2, 4, and 7 by overdoses of inhaled anesthetics ether. The skin of the treated wound area was removed for histological examination by hematoxylin–eosin staining. Observation and calculation of the number of new blood vessels formed were carried out using a binocular microscope equipped with an Optilab Advance V2 12,6MP camera in the five fields of view. Microscopic observations were performed at 40×, 100×, and 400× magnification. The number of new blood vessels formed was calculated by three observers. The number of new blood vessels were the only outcome measures used

### Statistical analysis

Two-way analysis of variance (ANOVA) was used to determine significant differences in angiogenesis among the treatment groups. Post hoc Tukey’s test was conducted to determine which groups showed significant differences. Statistical calculations were performed using statistical package for the social sciences (IBM SPSS Statistics 23) software at a confidence level of 95% (α < 0.05).

## Results

**Figure 3.**
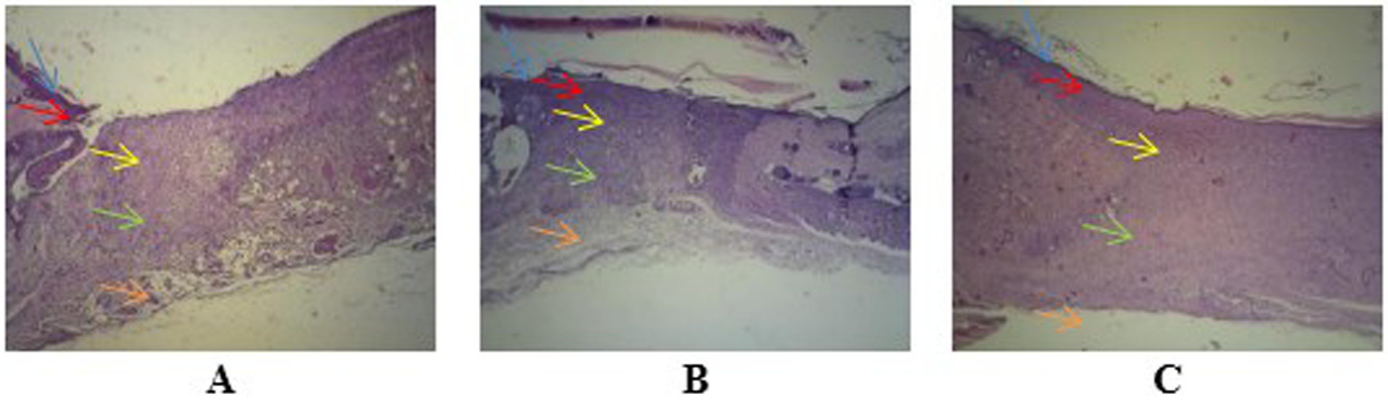
Wound healing with gel 96% on day two ( **A**), day four ( **B**), and day seven ( **C**), as determined using histological preparations with hematoxylin–eosin staining at 40× magnification. Blue arrows indicate the epithelium, red arrows indicate the epidermis, yellow arrows indicate the papillary stratum, green arrows indicate the reticular stratum, and orange arrows indicate adipose layers. Observation of angiogenesis was carried out on the papillary and reticular dermal layers of the stratum.

Observation at 40× magnification showed differences in the structure of wounds with healthy skin borders on days two, four, and seven at all perecentage of mucus ( Figure 3). Wounds observed on day two showed tissues filled with inflammatory cells without a surface epithelium. Wounds on day four revealed reductions in inflammatory tissue and a thin layer of connective tissue. The surface epithelium covering the wound was fairly thin. On day seven of healing, the wounds showed thick connective tissue formation and a surface epithelium layer clearly covering the wounded area. Inflammatory cells could not be clearly observed on day seven.

**Figure 4.**
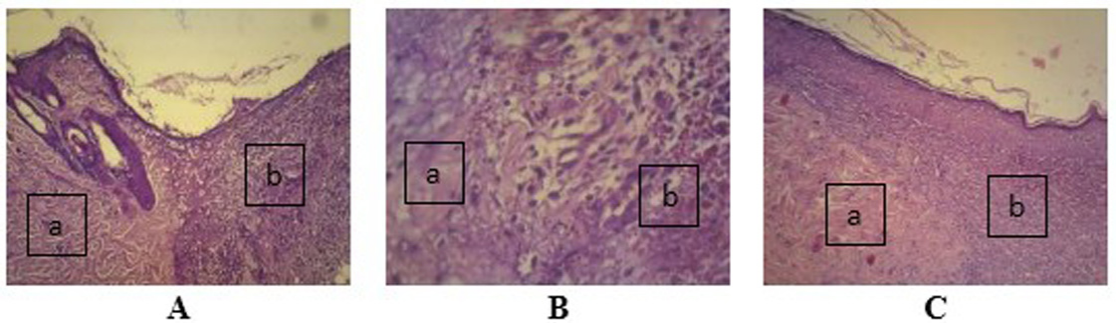
Wound healing with gel 96% on day two ( **A**), day four ( **B**), and day seven ( **C**), as determined using histological preparations with hematoxylin–eosin staining at 100× magnification. ( **a**) Healthy tissue and ( **b**) wound tissue.

Observation at 100× magnification was performed to determine relevant fields of view ( Figure 4). Angiogenesis was noted in the papillary and reticular strata. Visual fields for further observation were selected from five areas in these strata.

**Figure 5.**
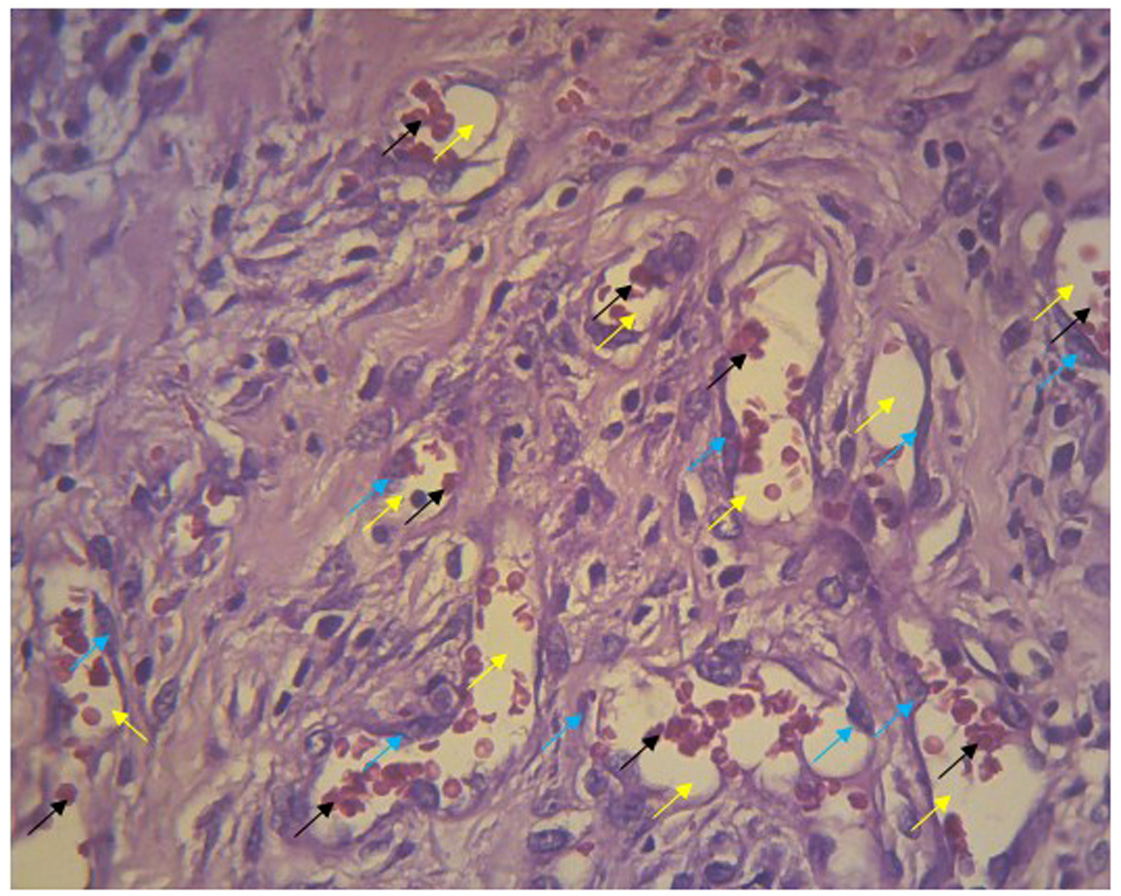
Blood vessels in the histologic preparation. The walls of lumen vessels (yellow arrows) are formed by endothelial cells (blue arrows) and contain erythrocytes (black arrows). This picture was taken from experiments with gel 96%.

The number of new blood vessels in the wounds was counted from five fields of view at 400× magnification. New blood vessels appeared in the lumen; the walls of these vessels were composed of endothelial cells and contained erythrocytes ( Figure 5). Endothelial cells at the edges of cell walls were purple in color, round, and flat. Erythrocytes appeared as red irregularly rounded cells without a nucleus.

**Figure 6.**
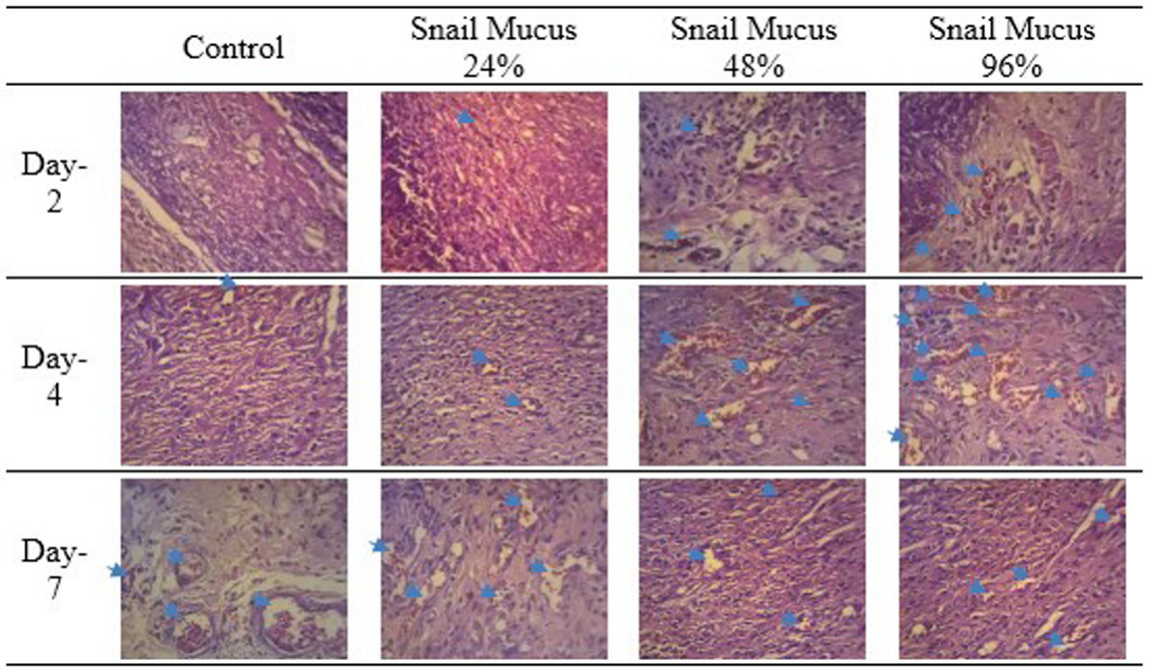
Angiogenesis in the wound area on days two, four, and seven, as observed from histological preparations with hematoxylin–eosin staining at 400× magnification. The greatest number of new blood vessels formed was observed on day four following the application of 96% snail mucus gel (p = 0.000).

**Figure 7.**
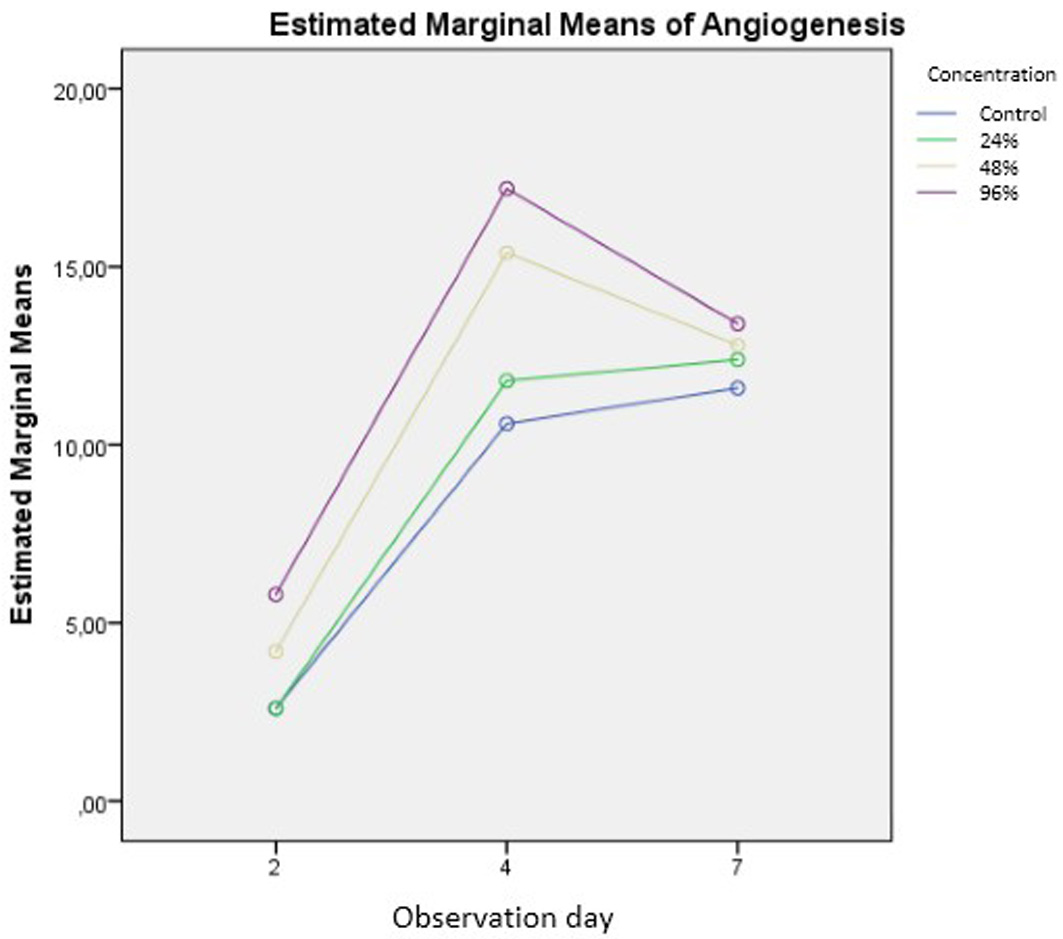
Graphic of the numbers of new blood vessels formed among treatment groups and days.

The number of new blood vessels formed are shown in Figure 6 and Table 1. The results of the calculations in Table 1 are depicted in Figure 7. The number of new blood vessels formed increased from day two to day four in all groups but was greatest in the 96% snail mucus treatment group (mean, 17.2). Whereas angiogenesis decreased from day four to day seven in the 96% and 48% snail mucus treatment groups, angiogenesis in the 24% snail mucus treatment and control groups increased over these days. The number of new blood vessels formed in the 96% snail mucus treatment group was consistently greater than those in the other treatment groups on each day assessed.

**Table 1.** Results of angiogenesis observations according to day and concentration.

Obsevation day	Fields of View	Mucus Snail Gel Concentration											
		Control			24%			48%			96%		
		Number/field	Total	Average	Number/ field	Total	Average	Number/ field	Total	Average	Number/ field	Total	Average
Day 2	1	3	13	2.6	3	13	2.6	4	21	4.2	5	29	5.8
	2	2			3			5			6		
	3	2			2			5			6		
	4	3			3			4			7		
	5	3			2			3			5		
Day 4	1	10	53	10.6	9	59	11.8	13	77	15.4	16	86	17.2
	2	11			10			16			16		
	3	9			15			17			18		
	4	13			13			14			19		
	5	10			12			17			17		
Day 7	1	13	58	11.6	12	62	12.4	12	64	12.8	14	67	13.4
	2	10			13			12			13		
	3	10			12			14			12		
	4	13			12			14			14		
	5	12			13			12			14		
Total average		41.33			44.67			54.00			60.67		

### Data analysis

Two-way ANOVA was used to determine significant differences in the extent of angiogenesis and number of observation days among the treatment groups. Differences in number of observation days, concentration, and the interaction of number of observation days and concentration were significant with 0.000, 0.000, and 0.001 (p < 0.05), respectively. These results demonstrate that each of the independent variables analyzed has a significant effect on angiogenesis.

Post hoc testing of the two-way ANOVA results was conducted using Tukey’s HSD test. The difference in the number of new blood vessels formed between the control group and the 24% snail mucus treatment group was not significant. All other groups showed significant differences in mean number of new blood vessels formed. These results indicate that treatment with 48% and 96% snail mucus has significant effects on angiogenesis during skin wound healing.

## Discussion

Wound healing is a biological process that involves complex interactions between cells, the extracellular matrix, and growth hormones. This process occurs in several phases, namely, hemostasis, inflammation, proliferation, and maturation ^26^. Angiogenesis is an important process in wound healing ^27^. The present study demonstrated that snail mucus can accelerate angiogenesis and wound healing. Prasojo *et al*. (2018) found that pure snail mucus without a carrier material can increase angiogenesis during wound healing compared with distilled water ^28^. Harti *et al*. (2018) showed that heparan sulfate stimulates VEGF. The study found that 5% and 100% snail mucus creams could accelerate wound healing by stimulating lymphocyte proliferation ^29^.

Xander and Toin (2013) ^30^ revealed that heparan sulfate is a proteoglycan that serves as a binder and storage unit for basic fibroblast growth factor (bFGF), which is secreted into the extracellular matrix. Heparan sulfate interacts with proangiogenic factors, such as fibroblast growth factor (FGF), VEGF, and platelet-derived growth factor (PDGF), on the surface of endothelial cells and causes these factors to bind to their corresponding receptors, thereby resulting in dimerization and various signaling processes. The extracellular matrix can release bFGF to stimulate inflammatory cell recruitment, fibroblast activation, and the formation of new blood vessels during injury ^31, 32^. This hormonal mechanism may also occur in the increase of angiogenesis in this study. However, research on this subject is still our next research project. In addition, a snail mucus gel formulation is also in our next project, so that this gel will have a more effective absorption rate and can be stored for a long time. A previous study indicated that snail mucus with chitosan as a membrane can accelerate wound healing through anti-inflammatory activity. Apriyanti *et al.* (2017) ^33^ also showed that 5% snail mucus gel could increase angiogenesis in alveolar bone during the healing of periodontitis in Wistar rats.

Angiogenesis is a complex process involving various cells, hormones, and extracellular components ^34^. Snail mucus contains proangiogenic glycosaminoglycans, heparan sulfate, heparin sulfate, and hyaluronic acid. These compounds can increase angiogenesis by triggering VEGF as the dominant angiogenetic growth factor against endothelial cells as blood vessel-forming cells ^22, 35, 36^.

Endothelial cell proliferation marks the beginning of angiogenesis. Endothelial cells grow, migrate, and then attach to the extracellular matrix, where they differentiate into new blood vessels ^37– 39^. Snail mucus can stimulate these processes remarkably. The results of this study demonstrated that the application of snail mucus could increase angiogenesis at all concentrations tested. The statistical analysis shows that the increase in angiogenesis is particularly significant at snail mucus concentrations of 48% and 96% (p = 0.000). Compared with the other groups, the 96% snail mucus treatment showed the greatest extent of angiogenesis ( Figure 7).

New blood vessels were formed on day two in all treatments. Wounds applied with snail mucus showed a greater number of new blood vessels formed compared with the control treatment (p = 0.000). This finding is consistent with the research of Bauer *et al*. (2005) ^15^, who found that the initial factor triggering angiogenesis is the damage that occurs in endothelial tubules following tissue damage. Tissue damage causes local hypoxia. The hypoxic state of the tissue becomes an angiogenic stimulator as growth factors and cytokines are released from inflammatory cells accumulated in the wound area during the previous inflammatory process. These factors stimulate the proliferation and invasiveness of vascular cells to promote blood vessel growth ^15, 16, 40^.

The most important mediators in the early phase of angiogenesis are VEGF, FGF, and PDGF ^41^. Heparan sulfate in snail mucus can interact with these factors on the surface of endothelial cells and enhance their ability to bind to their corresponding receptors, resulting in dimerization and various signaling processes ^30^. An adequate supply of nutrients, oxygen, and cells with essential functions in wound healing could hasten the wound healing process. Sufficient nutrition and oxygen are required for optimal wound healing. Angiogenesis provides a new vascular system that could deliver nutrients and oxygen to the wound area and enable wound healing. Cells necessary for wound healing, such as inflammatory cells, fibroblasts, and mesenchymal cells, which secrete various growth factors, may also be delivered to the wound site through this new vascular system ^42, 43^. Mature endothelial cells can then form new blood vessel walls in the wound area ^44^.

Day four of observation revealed the greatest number of new blood vessels formed in the 96% snail mucus treatment group. The proliferation phase of angiogenesis occurs on the fourth day after the initiation phase, which could explain the extent of angiogenesis observed on this day in the present study. Kalangi (2004) ^45^ stated that the proliferation phase begins with the degradation of old blood vessels by providing capillary shoot formation in hypoxic tissues to meet the nutritional and oxygen needs of parenchymal cells. These parenchymal cells secrete the most important proangiogenic growth factor, namely, VEGF-A. Then, there is a series of angiogenesis starting from (1) migration of endothelial cells distally from the original capillary vessels to stimulate angiogenesis; (2) proliferation of endothelial cells at the periphery of/distal to tubule formation; (3) stabilization of endothelial cells by interacting strongly with support cells, such as smooth muscle cells and pericytes; (4) maturation of endothelial cells via the formation of a lumen through intercellular and intracellular mechanisms, including the mobilization and proliferation of pericytes (from blood vessels) and smooth muscle cell (for large vessels) to support the endothelial wall and provide additional budding; (5) anastomosis with other endothelial buds and knot formation; and (6) development of circulation and adjustment of canals with arterial and venous segments ^45– 47^.

Glycosaminoglycans and heparan sulfate in snail mucus significantly increased (p = 0.031) the number of new blood vessels formed in the 96% snail mucus treatment group on day four. Glycosaminoglycans stabilize cell membranes, increase the synthesis of hyaluronic acid, a known anti-inflammatory agent, and accelerate angiogenesis; as such, these compounds have positive effects on wound healing ^48^. Angiogenesis can also be enhanced by the ability of snail mucus to bind divalent cations, such as copper(II) ^29^. Heparan sulfate is a proteogenizer that can bind and store bFGF, which is secreted by the extracellular matrix to stimulate the recruitment of inflammatory cells, fibroblast activation, and angiogenesis ^49^.

Compared with that on the day 4, the average number of new blood vessels formed in the 48% and 96% snail mucus treatment groups decreased on day 7. By contrast, the control and 24% snail mucus treatment groups revealed continuous increases in angiogenesis ( Table 1 and Figure 7) significantly (p = 0.000). This finding indicates that snail mucus not only increases the number of new blood vessels formed but also hastens the phases of angiogenesis. The decrease in number of new blood vessels formed on day seven in the 48% and 96% snail mucus treatment groups may be due to apoptosis. The number of new vessels formed is reduced until the density of blood vessels in the wound area returns to normal. This process is regulated by a selective apoptosis process that occurs simultaneously with the maturation of new blood vessels. Apoptosis refers to the automatic and programmed death of normal cells ^50– 52^.

According to a study conducted by Ricard *et al.* (2014), the glycosaminoglycans and hyaluronic acid in snail mucus could increase the activity of pericyte cells. Pericyte cells are multifunctional cells capable of maintaining capillary stability and protecting capillaries from negative signals. Some selective apoptosis processes are regulated by pericyte cells ^51, 53^. Pericytes are only present in newly formed blood vessels. Researchers believe that vessels without pericytes are susceptible to the influence of antiangiogenic agents ^54^. The apoptosis of blood vessels in a wound area decreases following the reduction of antiangiogenic factors ^55^. Mostafa (2014) ^56^ conducted a study on the effect of topical application of synthetic glycosaminoglycans on wound healing in mice and found an increase in wound closure speed; this study used synthetic glycosaminoglycans at a concentration of 2%. The glycosaminoglycans used in the present study are obtained naturally from snail mucus.

The most important mediators in angiogenesis are VEGF, FGF, and PDGF. This study did not examine hormonal levels of these factors to determine the effect of snail mucus on proangiogenic factors. Further research can be conducted to examine the effects of snail mucus on these factors and their corresponding receptors. In addition, a snail mucus gel formulation is also in our next project, so this gel will have a more effective absorption rate and can be stored for a long time.

## Conclusion

Different concentrations of snail mucus gel revealed different effects on angiogenesis during the healing of punch biopsy wounds on the back skin of Wistar rats. Compared with the control and 24% and 48% snail mucus treatment groups, the 96% snail mucus treatment group showed the greatest improvements in angiogenesis on day 4 (p = 0.00). Snail mucus concentration and day of observation showed interactive effects on angiogenesis during skin wound healing in Wistar rats (R = 0.946). Specifically, the higher the snail mucus concentration and the greater the number of observation days, the faster the wound healing process.

## Data availability

### Underlying data

Figshare: Table 1. Results of angiogenesis observations according to day and concentration ^56^. https://doi.org/10.6084/m9.figshare.13698871.v1

This project contains the following underlying data:

- Table 1. The results of the calculations in Table 1 are depicted in Figure 7. The number of new blood vessels formed increased from day 2 to day 4 in all groups but was greatest in the 96% snail mucus treatment group (mean, 17.2). Whereas angiogenesis decreased from day 4 to day 7 in the 96% and 48% snail mucus treatment groups, angiogenesis in the 24% snail mucus treatment and control groups increased over these days. The number of new blood vessels formed in the 96% snail mucus treatment group was consistently greater than those in the other treatment groups on each day assessed.

Figshare: Figure 1. Installation of excision wounds ^57^. https://doi.org/10.6084/m9.figshare.14045033.v1

This project contains the following data:

- JPG file of the installation of excision wounds on the rats. The skin on the back of a rat was folded and lifted by pinching the cranial and caudal skin between the thumb and forefinger. The rat was placed in the lateral position, and a biopsy punch was made through the folded skin (middle). Appearance of the resulting symmetrical and full-thickness wounds. No modifications have been made to this image.

Figshare. Figure 3. Wound healing with gel 98% on day two (A), day four (B), and day seven (C) ^58^. https://doi.org/10.6084/m9.figshare.14045075.v1

This project contains the following data:

- JPG file showing the wound healing on day 2. Determined using histological preparations with hematoxylin–eosin staining at 100× magnification. (a) Healthy tissue and (b) wound tissue. Arrow on the image was made by Microsoft Word 2013. No others modifications have been made to this image.

Figshare. Figure 4. Wound healing with gel 98% on day two (A), day four (B), and day seven (C) ^59^. https://doi.org/10.6084/m9.figshare.14045099

This project contains the following data:

- JPG file of wound healing with gel 98% on day two (A), day four (B), and day seven (C). Determined using histological preparations with hematoxylin–eosin staining at 100× magnification. (a) Healthy tissue and (b) wound tissue. Alphabet on the image was made by Microsoft Word 2013. No others modifications have been made to this image.

Figshare. Figure 5. Blood vessels in the histologic preparation ^60^. https://doi.org/10.6084/m9.figshare.14045309

This project contains the following data:

- JPG file of blood vessels in the histologic preparation. The walls of lumen vessels (yellow arrows) are formed by endothelial cells (blue arrows) and contain erythrocytes (black arrows). This picture was taken from experiments with gel 96%. Arrow on the image was made by Microsoft Word 2013. No others modifications have been made to this image.

Figshare. Figure 6. New blood vessels formation in the wound area on days two, four, and seven ^61^. https://doi.org/10.6084/m9.figshare.14045342

This project contains the following data:

- JPG file of angiogenesis in the wound area on days two, four, and seven. Observed from histological preparations with hematoxylin–eosin staining at 400× magnification. The greatest number of new blood vessels formed was observed on day four following the application of 96% snail mucus gel (p = 0.000). Grouping, naming, and arrow on the image were made by Microsoft Word 2013. No other modifications have been made to this image.

Figshare. Figure 7. Graphic of the numbers of new blood vessels formed among treatment groups and days ^62^. https://doi.org/10.6084/m9.figshare.14045351

This project contains the following data:

- JPG graphic of the numbers of new blood vessels formed among treatment groups and days No other modifications have been made to this image.

Figshare. Raw data of new blood vessels observation ^63^. https://doi.org/10.6084/m9.figshare.14045369

This project contains the following data:

- .xlxs file of the results of angiogenesis observations according to day and concentration.

Data are available under the terms of the Creative Commons Zero "No rights reserved" data waiver (CC BY 4.0 Public domain dedication).

### Extended data

Figshare. Figure 3A. Wound healing with gel 96% on day two ^64^. DOI: https://doi.org/10.6084/m9.figshare.14092499

Figshare. Figure 3B. Wound healing with gel 96% on day four as determined using histological preparations with hematoxylin–eosin staining at 40× magnification ^65^. DOI: https://doi.org/10.6084/m9.figshare.14092898

Figshare. Figure 3C. Wound healing with gel 96% on day seven, as determined using histological preparations with hematoxylin–eosin staining at 40× magnification ^66^. DOI: https://doi.org/10.6084/m9.figshare.14093035

Figshare. Figure 4A. Wound healing with gel 96% on day two as determined using histological preparations with hematoxylin–eosin staining at 100× magnification ^67^. DOI: https://doi.org/10.6084/m9.figshare.14093131

Figshare. Figure 4B. Wound healing with gel 96% on day four as determined using histological preparations with hematoxylin–eosin staining at 100× magnification ^68^. DOI: https://doi.org/10.6084/m9.figshare.14093227

Figshare. Figure 4C. Wound healing with gel 96% on day seven as determined using histological preparations with hematoxylin–eosin staining at 100× magnification ^69^. DOI: https://doi.org/10.6084/m9.figshare.14093289

Figshare. Figure 5. Blood vessels in the histologic preparation. The walls of lumen vessels (yellow arrows) are formed by endothelial cells (blue arrows) and contain erythrocytes (black arrows). This picture was taken from experiments with gel 96% ^70^. DOI: https://doi.org/10.6084/m9.figshare.14093357

Figshare. Figure 6 Day 2 24%. Angiogenesis in the wound area on day two with control as observed from histological preparations with hematoxylin–eosin staining at 400× magnification ^71^. DOI: https://doi.org/10.6084/m9.figshare.14093419

Figshare. Figure 6 Day 2 48%. Angiogenesis in the wound area on days two with 48% of snail mucus as observed from histological preparations with hematoxylin–eosin staining at 400× magnification ^72^. DOI: https://doi.org/10.6084/m9.figshare.14093731

Figshare. Figure 6 Day 2 96%. Angiogenesis in the wound area on days two with 96& of snail mucus as observed from histological preparations with hematoxylin–eosin staining at 400× magnification ^73^. DOI: https://doi.org/10.6084/m9.figshare.14093829

Figshare. Figure 6 Day 2 Control. Angiogenesis in the wound area on days two, four, and seven, as observed from histological preparations with hematoxylin–eosin staining at 400× magnification ^74^. DOI: https://doi.org/10.6084/m9.figshare.14093891

Figshare. Figure 6 Day 4 24%. Angiogenesis in the wound area on day four with 24% of snail mucus as observed from histological preparations with hematoxylin–eosin staining at 400× magnification ^75^. DOI: https://doi.org/10.6084/m9.figshare.14093935

Figshare. Figure 6 Day 4 48%. Angiogenesis in the wound area on day four with 48% of snail mucus as observed from histological preparations with hematoxylin–eosin staining at 400× magnification ^76^. DOI: https://doi.org/10.6084/m9.figshare.14094001

Figshare. Figure 6 Day 4 96%. Angiogenesis in the wound area on day four with 96% of snail mucus as observed from histological preparations with hematoxylin–eosin staining at 400× magnification ^77^. DOI: https://doi.org/10.6084/m9.figshare.14094623

Figshare. Figure 6 Day 4 Control. Angiogenesis in the wound area on day four with control as observed from histological preparations with hematoxylin–eosin staining at 400× magnification ^78^. DOI: https://doi.org/10.6084/m9.figshare.14094687

Figshare. Figure 6 Day 7 24%. Angiogenesis in the wound area on day seven with 24% of snail mucus as observed from histological preparations with hematoxylin–eosin staining at 400× magnification ^79^. DOI: https://doi.org/10.6084/m9.figshare.14094751

Figshare. Figure 6 Day 7 48%. Angiogenesis in the wound area on day seven with 48% of snail mucus as observed from histological preparations with hematoxylin–eosin staining at 400× magnification ^80^. DOI: https://doi.org/10.6084/m9.figshare.14094803

Figshare. Figure 6 Day 7 96%. Angiogenesis in the wound area on day seven with 96% of snail mucus as observed from histological preparations with hematoxylin–eosin staining at 400× magnification ^81^. DOI: https://doi.org/10.6084/m9.figshare.14094849

Figshare. Figure 6 Day 7 Control. Angiogenesis in the wound area on day seven with control as observed from histological preparations with hematoxylin–eosin staining at 400× magnification ^82^. DOI: https://doi.org/10.6084/m9.figshare.14094919
