# Supplementary material for: Effect of snail mucus on angiogenesis during wound healing
Source: F1000Res. 2021 May 21;10:181. Originally published 2021 Mar 5. [Version 2] doi: 10.12688/f1000research.51297.2 (PMC11190653; doi:10.12688/f1000research.51297.2)
Supplement: Peer review history

# Reviewer report: Reviewer response for version 2

**DOI:** 10.5256/f1000research.56728.r290498

**Published:** 2024-06-20

**Authors:** Jennifer Gubitosa

**Affiliations:** 1 Università di Bari, Bari, Italy

The Authors presented a paper entitled “Effect of snail mucus on angiogenesis during wound healing”.

 The work is interesting, and the manuscript is well written and quite well organized.

 However, in the following are some suggestions for improving the quality of the manuscript before its indexing.

 In the “Abstract”, when referring to the following sentence (“Snail mucus can bind to several factors”), it should be better to clarify the expressed  concept, specifying the meaning of the “binding”.

 In the “Introduction”, snail slime is not a material but a natural extract; please, check and correct it.

 In the “Materials” section, sub-section “Animals and group preparation”, please, check that the following sentence has been reported twice: “The rats were obtained from breeding by laboratory of pharmaceutical. Healthy male Wistar rats were adapted to cages for 3 days. The rat cages measured at least 40 cm long, 15 cm wide, and 10 cm high, and one cage housed one rat. The cages were covered with rice husks to achieve a stress-free environment for the rats, and the animals were provided food and water ad libitum.”

 In Figures 3, 4, 5, 6 the scale bars must be reported.

 Furthermore, it should be better to report the used microscope and the technique for obtaining the reported micrographs.

 The protocol followed for preparing the hybrid hydrogels CMC Na/Snail Slime extract should be better described.

 In the “Results” section, please, check the following sentences because it seems to be in contradiction: “Whereas angiogenesis decreased from day four to day seven in the 96% and 48% snail mucus treatment groups, angiogenesis in the 24% snail mucus treatment and control groups increased over these days. The number of new blood vessels formed in the 96% snail mucus treatment group was consistently greater than those in the other treatment groups on each day assessed.”

 In “Data availability” section, sub-section “underlying data”, check the “figshare figure 4” in which has been reported a 98% instead of 96%.

 In the “Discussion” section, please, check the following sentence (“proliferation of endothelial cells at the periphery of/distal to tubule formation”) and clarify it.

 Have the Authors preliminarily assessed the in vitro cytotoxicity of the explored snail slime % (24%, 48%, and 96%) on the endothelial cell lines?

Is the work clearly and accurately presented and does it cite the current literature?

Yes

If applicable, is the statistical analysis and its interpretation appropriate?

Partly

Are all the source data underlying the results available to ensure full reproducibility?

Partly

Is the study design appropriate and is the work technically sound?

Partly

Are the conclusions drawn adequately supported by the results?

Yes

Are sufficient details of methods and analysis provided to allow replication by others?

Partly

Reviewer Expertise:

Physical Chemistry, Polymeric materials, Nanotechnology, Pharmacy, Cosmetics, Natural extracts

I confirm that I have read this submission and believe that I have an appropriate level of expertise to confirm that it is of an acceptable scientific standard, however I have significant reservations, as outlined above.

# Reviewer report: Reviewer response for version 2

**DOI:** 10.5256/f1000research.56728.r85908

**Published:** 2022-05-17

**Authors:** Lidia Audrey Rocha Valadas, Francineudo Oliveira Chagas

**Affiliations:** 1 University of Buenos Aires, Buenos Aires, Argentina; 2 Federal University of Ceará, Fortaleza, Brazil

The suggestions were followed. I suggest acceptance for indexing.

Is the work clearly and accurately presented and does it cite the current literature?

Yes

If applicable, is the statistical analysis and its interpretation appropriate?

Yes

Are all the source data underlying the results available to ensure full reproducibility?

Yes

Is the study design appropriate and is the work technically sound?

Yes

Are the conclusions drawn adequately supported by the results?

Yes

Are sufficient details of methods and analysis provided to allow replication by others?

Yes

Reviewer Expertise:

pharmacology

We confirm that we have read this submission and believe that we have an appropriate level of expertise to confirm that it is of an acceptable scientific standard.

# Reviewer report: Reviewer response for version 1

**DOI:** 10.5256/f1000research.54450.r82617

**Published:** 2021-05-04

**Authors:** Jike Lu

**Affiliations:** 1 School of Life Sciences, Zhengzhou University, Zhengzhou, Henan, China

- This is an interesting study on the angiogenesis of crude snail mucus without purification.
- About the “Snail mucus gel preparation”, how to store the prepared mucus, or how to ensure the stability of snail mucus during the animal experiments in seven days?
- About the animal experiments, a positive control group is recommended. How to ensure that the mucus (1mL applied on each wound) does not flow out or affect other groups due to animal movement? Description about the animal cage and environment is repeated.
- Figure 2, Group 1-3 shows similar photos. It is suggested to list the wound photos at different days to observe the wound healing.
- The work is descriptive and needs more mechanisms of research.

Is the work clearly and accurately presented and does it cite the current literature?

Partly

If applicable, is the statistical analysis and its interpretation appropriate?

Yes

Are all the source data underlying the results available to ensure full reproducibility?

Yes

Is the study design appropriate and is the work technically sound?

Partly

Are the conclusions drawn adequately supported by the results?

Yes

Are sufficient details of methods and analysis provided to allow replication by others?

Yes

Reviewer Expertise:

Natural product chemistry.

I confirm that I have read this submission and believe that I have an appropriate level of expertise to confirm that it is of an acceptable scientific standard, however I have significant reservations, as outlined above.

## References

[1] : Wound-healing activity of glycoproteins from white jade snail (Achatina fulica) on experimentally burned mice. *Int J Biol Macromol* .2021;175: 10.1016/j.ijbiomac.2021.01.193 313-321 10.1016/j.ijbiomac.2021.01.193 33539960

# Response

**Published:** 2021-05-06

**Authors:** Yosaphat Bayu Rosanto

**Affiliations:** Universitas Gadjah Mada, Indonesia

- The mucus was diluted with CMC-Na to obtain gels with mucus concentrations of 24%, 48%, and 96% (w/w). This concentration produces a gel that is thick enough and does not flow out of the wound. The prepared mucus were stored in polypropylene conical tubes, covered with aluminum foil and placed in the refrigerator at 4 °C. This storage method preserved the angiogenic effect of the mucus. This was evidenced by the number of new blood vessels that were still significantly affected by the results of the study on day 7. Temperature and light could affect the stability of the mucus so that the used of opaque aluminum foil and cold temperatures could maintain product quality well.
- The minimum distance between wounds was 5 mm. The distance between the wounds in this study was made more than 2 cm so that it does not allow mucus to flow out or affect other groups due to animal movement.
- Positive control was not used in this study. The negative control showed a natural angiogenesis process for comparison with the mucus treatment group.
- The total rats in each groups are described in Figure 2. (Disclaimer: this image is only an illustration to explain the study grouping, not a photo of each mouse used in this study group).

# Reviewer report: Reviewer response for version 1

**DOI:** 10.5256/f1000research.54450.r81121

**Published:** 2021-03-22

**Authors:** Lidia Audrey Rocha Valadas

**Affiliations:** 1 University of Buenos Aires, Buenos Aires, Argentina

- In general the work is interesting and well designed and with appropriate methodology and details that guarantee reproducibility.
- The introduction is clear but i suggest to add the importance of the new patent on the field.
- I suggest to make the conclusion more concise, without statistical data, on this topic you just need to answer the objective of the study.

Is the work clearly and accurately presented and does it cite the current literature?

Yes

If applicable, is the statistical analysis and its interpretation appropriate?

Yes

Are all the source data underlying the results available to ensure full reproducibility?

Yes

Is the study design appropriate and is the work technically sound?

Yes

Are the conclusions drawn adequately supported by the results?

Yes

Are sufficient details of methods and analysis provided to allow replication by others?

Yes

Reviewer Expertise:

pharmacology

I confirm that I have read this submission and believe that I have an appropriate level of expertise to confirm that it is of an acceptable scientific standard.
